# Role of Tocilizumab in Down Regulating sCD163 Plasmatic Levels in a Cohort of COVID-19 Patients

**DOI:** 10.3389/fimmu.2022.871592

**Published:** 2022-04-04

**Authors:** Raffaella Marocco, Anna Carraro, Maria Antonella Zingaropoli, Parni Nijhawan, Eeva Tortellini, Mariasilvia Guardiani, Fabio Mengoni, Paola Zuccalà, Valeria Belvisi, Blerta Kertusha, Alberico Parente, Cosmo Del Borgo, Vincenzo Vullo, Maria Rosa Ciardi, Claudio Maria Mastroianni, Miriam Lichtner

**Affiliations:** ^1^ Infectious Diseases Unit, Santa Maria (SM) Goretti Hospital, Sapienza University of Rome, Latina, Italy; ^2^ Department of Public Health and Infectious Diseases, Sapienza University of Rome, Rome, Italy

**Keywords:** monocytes/macrophages, sCD163, IL-6, tocilizumab, SARS-CoV-2, ELISA

## Abstract

**Background:**

CD163, a haptoglobin-hemoglobin scavenger receptor mostly expressed by monocytes and macrophages, is involved in the regulation of inflammatory processes. Following proteolytic cleavage after pro-inflammatory stimulation, CD163 is shed from the cell surface and its soluble form in plasma, sCD163, is a biomarker of monocyte/macrophage lineage activation.

The assessment of sCD163 plasmatic levels in an early stage of the disease could have clinical utility in predicting the severity of COVID-19 pneumonia. The use of tocilizumab (monoclonal antibody anti-IL-6 receptor) in COVID-19 patients reduces lethality rate at 30 days. The aim of the study was to investigate the effect of tocilizumab on sCD163 plasmatic levels in a cohort of COVID-19 patients.

**Methods:**

In COVID-19 patients, on hospital admission (T0), after 7 days from hospitalization (T7) and after 45 days from discharge (T45) sCD163 plasmatic levels were evaluated, along with other laboratory parameters. COVID-19 patients were stratified into tocilizumab (TCZ) and non-tocilizumab (non-TCZ) groups. TCZ group was further divided into responder (R) and non-responder (NR) groups. Patients who died or required mechanical ventilation were defined as NR. As control group, healthy donors (HD) were enrolled.

**Results:**

Seventy COVID-19 patients and 47 HD were enrolled. At T0, sCD163 plasmatic levels were higher in COVID-19 patients compared to HD (p<0.0001) and the longitudinal evaluation showed a reduction in sCD163 plasmatic levels at T7 compared to T0 (p=0.0211). At T0, both TCZ and non-TCZ groups showed higher sCD163 plasmatic levels compared to HD (p<0.0001 and p=0.0147, respectively). At T7, the longitudinal evaluation showed a significant reduction in sCD163 plasmatic levels (p=0.0030) only in the TCZ group, reaching levels comparable to those of HD. Conversely, not statistically significance in non-TCZ group was observed and, at T7, a statistically significance was found comparing non-TCZ group to HD (p=0.0019). At T0, R and NR groups showed not statistically significance in sCD163 plasmatic levels and both groups showed higher levels compared to HD (p=0.0001 and p=0.0340, respectively). The longitudinal evaluation showed significant reductions in both groups (R: p=0.0356; NR: p=0.0273) independently of the outcome. After 45 days of follow-up sCD163 plasmatic levels remain stable.

**Conclusion:**

sCD163 plasmatic levels are increased in COVID-19 pneumonia and is efficiently down-regulated by tocilizumab treatment regardless of the clinical outcome.

## Introduction

The current COVID-19 pandemic which originated in December 2019 and is still actively spreading at a rapid and mass scale has managed to grab enormous attention from researchers globally providing great insights into a deeper analysis emphasizing on the SARS-COV-2 genome, immunopathogenesis and vaccine development (1, 2). One of the strongest components of immune response studies are cytokines, immune cells and blood biomarkers (2–5).

Cytokines are low molecular weight immunomodulating proteins that operate by coordinating communication between cells and cooperating among inflammation and immunity (3). In this context, circulating cytokines can play an important role as biomarkers and can be used in the diagnosis, and response to treatment in infectious diseases (6). The proliferation and activation of monocytes/macrophages is the most significant step in the initiation of the immunopathogenesis of a wide range of infections and is thought to contribute to the pathogenesis of COVID-19 pneumonia concomitantly with the cytokine storm (4, 7).

In particular, soluble CD163 (sCD163) is a soluble form of CD163, a protein biomarker for the activation of monocyte/macrophage cell lineage which basically is a scavenger receptor for hemoglobin haptoglobin complex possessing very high affinity (8). This soluble inflammatory cytokine is generally found in the plasma, serum, and cerebrospinal fluid of all healthy individuals in a normal range (9). An upregulation in the concentration of sCD163 is indicative of a strong immune response in individuals suffering from many viral and bacterial infections such as HCV, HIV, CMV, HPV (10–14). sCD163 is generally considered to be a result of proteolytic cleavage of monocyte bound CD163 by matrix metalloproteinases (MMPs) (9). A high oxidative stress is supposed to be a driving force for the release of sCD163. As a result of the shedding, during inflammation and activation of macrophages, the extracellular portion of CD163 circulates in the blood as sCD163 (9). Elevated CD163 expression on alveolar macrophages has been reported in patients with chronic obstructive pulmonary disease (BPCO) and in idiopathic pulmonary fibrosis (9). Several authors reported an increment of sCD163 plasmatic levels with worsening COVID-19 severity, underlining a preponderant role for monocyte-macrophage activation in the development of immunopathology of COVID-19 patients (6, 15–17).

An ongoing decline in sCD163 plasmatic levels with respect to effective therapy has been reported in other viral infections (18). Several immunomodulator compounds have been tested against COVID-19 pneumonia by disrupting the phenomenon of cytokine storm (4, 19, 20). Moreover, specific immune modulators include anti-IL-6 and IL-1 receptor antagonists (tocilizumab, sarilumab, anakirna) and Janus kinase (JAK1/JAK2) inhibitors, that determine a dose-dependent inhibition of IL-6-induced STAT3 phosphorylation (baricitinib) (20–26).

Although many proinflammatory cytokines are involved in cytokine release syndrome (CRS), interleukin-6 (IL-6) is the most important one (4, 19). Anti-IL-6 agents have been proposed as a promising treatment regimen for COVID-19 pneumonia (27). Tocilizumab is a humanized monoclonal antibody that can target both membrane-bound and soluble forms of the IL-6 receptor, and several studies have evaluated its efficacy in treating severe COVID-19 pneumonia (19, 20, 27, 28). The effectiveness of tocilizumab in down regulating the concentration of cytokines such as IL-6, IL-17 is well studied and understood (29).

The aim of this study was to investigate the effect of tocilizumab in sCD163 plasmatic level at different time points in a cohort of hospitalized COVID-19 patients.

## Materials and Methods

### Study Design and Participants

From March 2020 to June 2020, patients with COVID-19 pneumonia admitted to S.M Goretti Hospital of Latina, were enrolled. COVID-19 related pneumonia was diagnosed by computed tomography (CT scan) of the chest associated with SARS-CoV-2 RNA detection from a nasopharyngeal swab through a commercial reverse transcription polymerase chain reaction (RT-PCR) kit, following manufacturer’s instructions (RealStar^®^ SARS-CoV-2 Altona Diagnostic, Germany).

On hospital admission, clinical information, and routine laboratory exams, including demographics, respiratory parameters with arterial oxygen partial pressure/fraction of inspired oxygen (PaO_2_/FiO_2_) ratio, lactate dehydrogenase (LDH), C-reactive protein (CRP), ferritin, D-dimer, blood neutrophil, lymphocyte and monocyte absolute counts were collected.

All patients have received as standard of care (SoC) a combination of lopinavir/ritonavir, hydroxychloroquine, steroids (methylprednisolone), low-weight molecular heparin (LWMH) as prophylaxis, and oxygen support depending on degree of respiratory failure.

Tocilizumab was administered intravenously (8 mg/kg) according to availability and following physician decision.

According to tocilizumab treatment, COVID-19 patients were stratified into tocilizumab (TCZ) and non-tocilizumab (non-TCZ) groups. Moreover, TCZ group was further stratified into responders (R) for those who responded to therapy and non-responders (NR) for those who failed to respond to tocilizumab therapy. Failure was defined when death or intubation occurred after treatment.

Finally, as control group, healthy donors (HD) matched for age and sex distribution, without any symptom, and with a negative nasopharyngeal swab for SARS-CoV-2 RNA detection and undetectable anti-SARS-CoV-2 specific IgG, were enrolled.

### Measurement of sCD163 Plasmatic Levels

On hospital admission, during routine clinical testing, peripheral whole blood samples, collected in heparin tubes, were drawn in hospitalized COVID-19 patients at different time-points: on hospital admission (T0), after 7 days from hospitalization (T7) and at follow-up after 30-45 days discharge (T45).

Plasma was obtained after centrifugation and immediately stored at -80°C until use. sCD163 plasmatic level was quantified using enzyme-linked immunosorbent assay (ELISA) kits (Quantikine, R&D Systems, Minneapolis, Minnesota, USA). Standard curves and samples were tested in duplicate. The limit of detection for sCD163 was 0.177 ng/ml.

### Statistical Analysis

All statistical analyses were performed using GraphPad Prism v.9 software and two-tailed p ≤ 0.05 was considered statistically significant. Values are represented as median and interquartile range (IQR).

The nonparametric comparative Mann-Whitney test and the nonparametric Kruskal-Wallis test with Dunn**’**s post-test were used for comparing medians between groups. Longitudinal evaluation of sCD163 plasmatic levels was performed using the nonparametric Wilcoxon test. Spearman rank correlation analysis was used to assess the relation between clinical and laboratory data and sCD163 plasmatic levels (Spearman coefficient [ρ] and statistical significance [p] are reported in the graphics). Linear correlation was evaluated using the regression test.

## Results

### Demographic and Clinical Laboratory Parameters of Study Population

Seventy hospitalized COVID-19 patients (41 males and 29 females, median age [IQR] of 66 [54-77] years) and 47 HD (24 males and 23 females, median age [IQR] of 61 [55-67] years) were enrolled. None of the COVID-19 patients enrolled in the present study was infected with HIV.

According to chest CT scan findings, all COVID-19 patients showed sign of interstitial pneumonia. Concerning comorbidities, 66% of COVID-19 patients had at least one coexisting illness and the prevalent were hypertension (41.4%), cardiovascular disease (29.0%), and diabetes (26.0%). Among all COVID-19 patients, 20% died due to worsening of their condition (**Table 1**).

**Table 1:** Demographic and clinical features of study population on hospital admission.

	COVID-19 (n = 70)	HD (n = 47)	TCZ (n = 45)	non-TCZ (n = 25)	p value*
**Male/Female**	41/29	24/23	28/17	13/12	ns
**Age, median (IQR) years**	66 (53–77)	61 (55-67)	64 (54-76)	69 (51-81)	ns
**ARDS/non-ARDS**	34/36	–	13/32	21/4	p<0.0001
**Deaths/Alive**	14/56	–	10/35	4/21	ns
**Comorbidities**	46	–	27	19	ns
Others	28	–	16	12	ns
Hypertension	29	–	19	10	ns
Cardiovascular	20	–	9	11	ns
Diabetes	17	–	8	9	ns
Respiratory	8	–	6	2	ns
Neoplasia	6	–	4	2	ns
Renal	3	–	2	1	ns
**Laboratory findings**					
Neutrophils (x10^9^/L)	4.4 (3.0-6.4)	–	4.5 (3.0-6.4)	4.4 (3.1-6.3)	ns
Lymphocytes (x10^9^/L)	0.9 (0.6-1.4)	–	0.8 (0.6-1.1)	1.4 (0.9-1.9)	p=0.0009
Monocytes (x10^9^/L)	0.4 (0.3-0.7)	–	0.5 (0.3-0.7)	0.4 (0.2-0.8)	ns
NLR	4.5 (2.6-7.5)	–	5.8 (3.0-9.1)	3.3 (2.0-6.1)	p=0.0185
CRP (mg/dl)	4.1 (0.7-10.6)	–	5.4 (3.1-12.7)	0.7 (0.2-6.4)	p=0.0007
D-dimer (µg/ml)	0.8 (0.3-1.5)	–	1.0 (0.4-1.6)	0.5 (0.3-4.1)	ns
Ferritin (ng/ml)	394 (179-653)	–	538 (363-1111)	179 (107-389)	p=0.0018
LDH (U/L)	272 (224-380)	–	301 (257-425)	220 (185.3-252)	p<0.0001

TCZ, tocilizumab; n, number; IQR, interquartile range; ARDS, Acute distress respiratory syndrome; NRL, neutrophil/lymphocyte ratio; CRP, C-reactive protein; LDH, lactate dehydrogenase; ns, not significant. The 2-tailed X^2^ test or Fisher’s exact test was used for comparing proportions between TCZ and non-TCZ groups. The nonparametric comparative Mann-Whitney test was used to compare medians between TCZ and non-TCZ groups. *The differences were evaluated between TCZ and non-TCZ groups.

On hospital admission, median (IQR) values of plasmatic ferritin (394 [179-653] ng/mL), LDH (272 [224-380]) U/L), D-dimer (0.8 [0.3-1.5] µg/mL) and CRP (4.1 [0.7-10.6] mg/mL) were higher in COVID-19 patients compared to the normal range (**Table 1**).

Overall, 34 COVID-19 patients developed a severe form of COVID-19 pneumonia with acute respiratory distress syndrome (ARDS group) while 36 showed a COVID-19 pneumonia without ARDS (non-ARDS group) (**Table 1**).

### Longitudinal Evaluation of sCD163 in COVID-19 Patients

**Figure 1:**
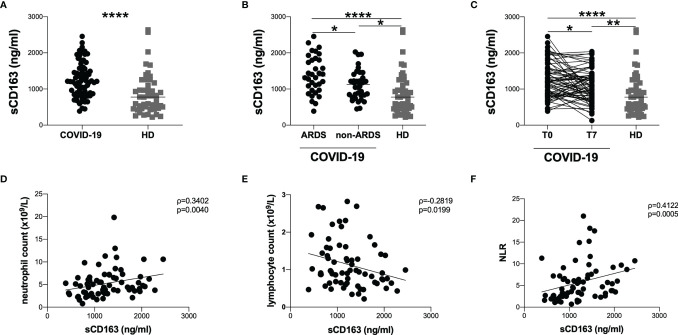
Evaluation of sCD163 plasmatic levels and correlations with clinical data. **(A)** sCD163 plasmatic levels were evaluated in 70 COVID-19 patients and 47 HD. The differences were evaluated using the nonparametric Mann-Whitney test. Data are shown as median (lines). **(B)** sCD163 plasmatic levels were evaluated in 34 patients with ARDS (ARDS group) and 36 patients without ARDS (non-ARDS group) using the nonparametric Mann-Whitney test. Both ARDS and non-ARDS groups were compared to HD using the nonparametric Kruskal-Wallis test with Dunn’s post-test. Data are shown as median (lines). **(C)** sCD163 plasmatic levels were longitudinal evaluated in 70 COVID-19 patients at two time-points: at T0 (on hospital admission) and T7 (after seven days from hospital admission) using Wilcoxon test. Both T0 and T7 were compared to HD using the nonparametric Kruskal-Wallis test with Dunn**’**s post-test. Data are shown as median (lines). **(D)** Positive correlation between sCD163 plasmatic levels and absolute neutrophil count on 70 COVID-19 patients. Linear correlation was evaluated by using the regression test, R^2^ = 0.0696, p=0.0273. **(E)** Negative correlation between sCD163 plasmatic levels and absolute lymphocytes count on 70 COVID-19 patients. Linear correlation was evaluated by using the regression test, R^2^ = 0.0702, p=0.0290. **(F)** Positive correlation between sCD163 plasmatic levels and neutrophil/lymphocyte ratio (NLR) on 70 COVID-19 patients. Linear correlation was evaluated by using the regression test, R^2^ = 0.0843 p=0.0171. All correlations were performed using Spearman test. Spearman coefficient (ρ) and statistical significance (p) are reported in the graphics. **** p> 0.0001; **0.01 < p < 0.001; *0.05 < p <0.01.

Overall, sCD163 plasmatic levels were higher in COVID-19 patients compared to HD (1209 [863-1563] and 777 [458-1169], respectively; p<0.0001) (**Figure 1A**) as well as in ARDS group compared to non-ARDS one (1359 [967-1814] and 1126 [819-1381], respectively; p=0.0230) (**Figure 1B**). Both ARDS and non-ARDS groups showed higher sCD163 plasmatic levels compared to HD (p<0.0001 and p=0.0154, respectively) (**Figure 1B**).

The longitudinal evaluation performed in 70 COVID-19 patients showed a significant decrease in sCD163 plasmatic levels at T7 compared to T0 (1060 [766-1350] and 1209 [823-1563], respectively; p=0.0211). Both at T0 and T7 COVID-19 patients showed significantly higher sCD163 plasmatic levels compared to HD (p<0.0001 and p=0.0071, respectively) (**Figure 1C**).

Considering all COVID-19 patients, at T0 we observed positive correlations between sCD163 plasmatic levels and absolute neutrophil count (ρ=0.3402, p=0.0040) as well as between sCD163 plasmatic levels and neutrophil/lymphocytes ratio (ρ=0.4122, p=0.0005) (**Figures 1D** **, F**). Conversely, a negative correlation between sCD163 plasmatic levels and absolute lymphocyte count was found (ρ=-0.2819, p=0.0199) (**Figure 1E**). There was no correlation between monocyte absolute count and sCD163 plasmatic levels. Moreover, no association between sCD163 plasmatic levels and age of the COVID-19 patients was observed nor differences between males and females.

### Evaluation of sCD163 According to Tocilizumab Treatment

To evaluate if the longitudinal decrease in sCD163 plasmatic levels observed was due to tocilizumab treatment, COVID-19 patients were stratified according to tocilizumab treatment.

Forty-five COVID-19 patients were treated with tocilizumab (TCZ group) while 25 were not treated with tocilizumab (non-TCZ group). No statistically difference was observed between TCZ and non-TCZ groups concerning age, gender, and coexisting illness. On hospital admission, TCZ group showed significantly lower absolute lymphocyte count (p=0.0009) and higher plasmatic levels of CRP (p=0.0007), LDH (p<0.0001), ferritin (p=0.0018) compared to non-TCZ group (**Table 1**). A higher percentage of deaths in non-TCZ group compared to TCZ one was observed, although not statistically significant (28.0% and 15.6%, respectively). Finally, a significantly higher percentage of patients who developed ARDS during hospitalization was found in non-TCZ group compared to TCZ one (84.0% and 16%, p<0.0001) (**Table 1**).

**Figure 2:**
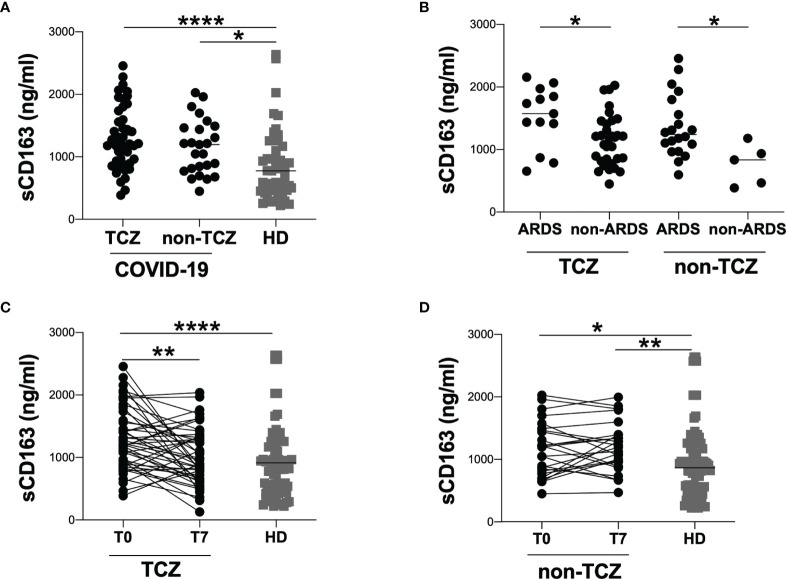
Evaluation of sCD163 plasmatic levels in tocilizumab and non-tocilizumab groups. **(A)** sCD163 plasmatic levels were evaluated in 45 tocilizumab treated patients (TCZ) and 25 tocilizumab untreated patients (non-TCZ). The differences were evaluated using the nonparametric Mann-Whitney test. Data are shown as median (lines). Both TCZ and non-TCZ groups were compared to HD using the nonparametric Kruskal-Wallis test with Dunn’s post-test. Data are shown as median (lines). **(B)** sCD163 plasmatic levels were evaluated in tocilizumab treated (TCZ) and tocilizumab untreated (non-TCZ) patients stratified according to the development of ARDS. The differences were evaluated using the nonparametric Mann-Whitney test. Data are shown as median (lines). **(C)** sCD163 plasmatic levels were longitudinal evaluated in 45 tocilizumab treated patients at two time-points: at T0 (on hospital admission) and T7 (after seven days from hospital admission) using Wilcoxon test. Both T0 and T7 were compared to HD using the nonparametric Kruskal-Wallis test with Dunn’s post-test. Data are shown as median (lines). **(D)** sCD163 plasmatic levels were longitudinal evaluated in 25 tocilizumab treated patients at two time-points: at T0 (on hospital admission) and T7 (after seven days from hospital admission) using Wilcoxon test. Both T0 and T7 were compared to HD using the nonparametric Kruskal-Wallis test with Dunn**’**s post-test. Data are shown as median (lines). ****p > 0.0001; **0.01 < p < 0.001; *0.05 < p <0.01.

At T0, comparing TCZ and non-TCZ group no statistically significant difference in sCD163 plasmatic levels was observed (1211 [913-1664] and 1195 [793-1478], respectively) (**Figure 2A**). Both TCZ and non-TCZ groups showed higher sCD163 plasmatic levels compared to HD (p<0.0001 and p=0.0147, respectively) (**Figure 2A**).

Stratifying TCZ and non-TCZ groups according to the development of ARDS, higher sCD163 plasmatic levels were observed in ARDS groups compared to respectively non-ARDS groups (TCZ group: 1573 [1141-1911] and 1185 [822-1443], respectively; p=0.0178. non-TCZ group: 1240 [998-1739] and 835 [426-1056], respectively; p=0.0122) (**Figure 2B**). No significant differences were observed comparing ARDS group from TCZ group to ARDS group from non-TCZ one as well as comparing non-ARDS group from TCZ group to non-ARDS group from non-TCZ one (**Figure 2B**).

At T7, the longitudinal evaluation in TCZ group showed a significant reduction of sCD163 plasmatic levels compared to T0 (1211 [913-1664] and 895 [657-1338], respectively; p=0.0030) (**Figure 2C**). Moreover, no significant difference was found comparing T7 to HD (**Figure 2C**).

Regarding non-TCZ group, no significant difference in sCD163 plasmatic levels was observed comparing T0 to T7, while a significant difference in sCD163 plasmatic levels was found comparing T7 to HD (1196 [793-1478] and 1192 [921-1395], respectively; p=0.0019) (**Figure 2D**).

### Evaluation of sCD163 According to Response to the Therapy

According to response to therapy, TCZ group was further stratified into R (n=35), who recovered after therapy, and NR (n=10), who died because of COVID-19 due to worsening of condition even after therapy.

**Figure 3:**
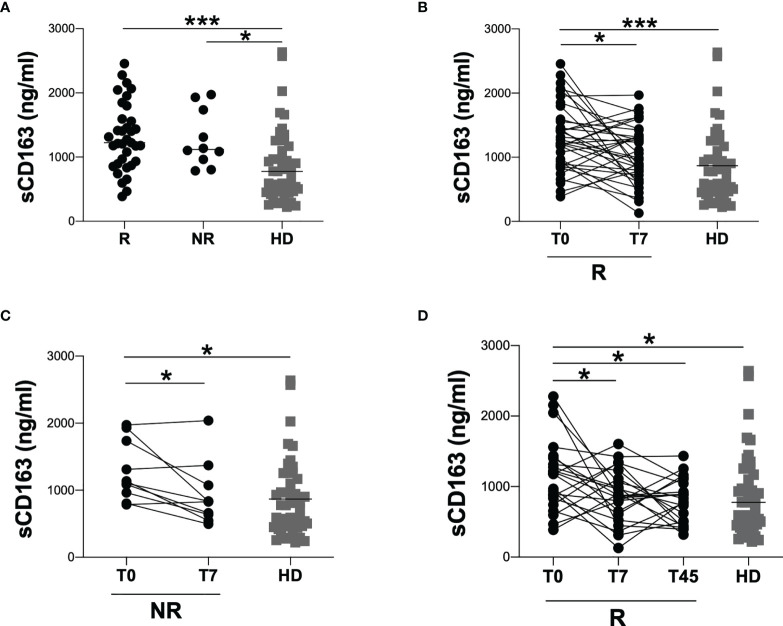
Evaluation of sCD163 plasmatic levels according to response to tocilizumab treatment. **(A)** sCD163 plasmatic levels were evaluated in 35 responder (R) and 10 non-responder (NR) patients. The differences were evaluated using the nonparametric Mann-Whitney test. Data are shown as median (lines). Both R and NR groups were compared to HD using the nonparametric Kruskal-Wallis test with Dunn’s post-test. Data are shown as median (lines). **(B)** sCD163 plasmatic levels were longitudinal evaluated in 35 responder (R) patients at two time-points: at T0 (on hospital admission) and T7 (after seven days from hospital admission) using Wilcoxon test. Both T0 and T7 were compared to HD using the nonparametric Kruskal-Wallis test with Dunn’s post-test. Data are shown as median (lines). **(C)** sCD163 plasmatic levels were longitudinal evaluated in 10 non-responder (NR) patients at two time-points: at T0 (on hospital admission) and T7 (after seven days from hospital admission) using Wilcoxon test. Both T0 and T7 were compared to HD using the nonparametric Kruskal-Wallis test with Dunn’s post-test. Data are shown as median (lines). **(D)** sCD163 plasmatic levels were longitudinal evaluated in 22 responder (R) patients at three time-points: at T0 (on hospital admission), T7 (after seven days from hospital admission) and T45 (30-45 days from discharge) using Friedman test with Dunn’s post-test. Each time-point (T0, T7 and T45) was compared to HD using the nonparametric Kruskal-Wallis test with Dunn’s post-test. Data are shown as median (lines). ***0.0001<p<0.001; *0.01<p<0.05.

At T0, the evaluation of sCD163 plasmatic levels showed no significant difference in sCD163 plasmatic levels comparing R and NR groups (1224 [893-1593] and 1119 [924-1784], respectively) (**Figure 3A**). However, at T0, both R and NR groups showed significantly higher sCD163 levels compared to HD (p=0.0001 and p=0.0340, respectively) (**Figure 3A**).

At T7, the longitudinal evaluation of sCD163 plasmatic levels in R and NR group showed a significant reduction of sCD163 plasmatic levels compared to T0 (R group: 1224 [893-1593] and 988 [722-1343], respectively; p=0.0356. NR group: 1119 [924-1784] and 831 [615-1149], respectively; p=0.0273) (**Figures 3B, C**). At T7, both NR and R groups showed no significant difference compared to HD (**Figures 3B** **, C**).

Finally, for 22 COVID-19 patients of R group, a further evaluation of sCD163 plasmatic levels was performed at T45 showing a significant reduction compared to T0 (T0: 1179 [812-1412], T7: 868 [588-1141] and T45: 807 [486-1059], p=0.0475). At T45 COVID-19 patients showed sCD163 plasmatic levels comparable to those of HD (**Figure 3D**).

## Discussion

Here, we assessed the effect of tocilizumab on sCD163 plasmatic levels in a cohort of hospitalized COVID-19 patients evaluating the dynamic changes between hospital admission and after 7 days from hospitalization. Moreover, in a subgroup of COVID-19 patients we evaluated sCD163 plasmatic levels after 45 days from discharge.

Several studies have described the evaluation of sCD163 plasmatic levels at an early stage of the disease and have demonstrated its utility in predicting the severity of COVID-19 pneumonia (6, 15, 16). Although sCD163 plasmatic level is not a routine evaluation in COVID-19 patients, all these reports suggest that sCD163 plasmatic levels could represent a useful and easily assessable biomarker of disease progression underlining its clinical utility.

Different immunomodulator compounds explicate their effects disrupting the phenomenon of the cytokine storm involved in the immunopathogenesis of COVID-19 (15–19). Currently, anti-IL-6 agents have been proposed as a promising therapy for COVID-19 (16, 20). Specifically, tocilizumab, an anti-IL-6 receptor monoclonal antibody, has been found to be effective in regulating the levels of cytokines such as IL-6 and IL-17 and its administration in COVID-19 patients has been shown to reduce the lethality rate at 30 days (15, 21).

The idea that in COVID-19 patients tocilizumab may suppress the cytokine storm by decreasing the activity of IL-6, is corroborated by the findings of Zarinsefat et al., who speculated on the mechanistic/biologic effects of this drug on immune system cells using an *in vitro* cytokine storm model of peripheral blood mononuclear cells (PBMC) (30). Specifically, the authors comparing single-cell RNA sequencing (scRNA-seq) of stimulated PBMC from kidney transplant recipients with subclinical rejection with and without tocilizumab treatment, showed that tocilizumab-treated PBMC had reduced expression of inflammatory-mediated genes and biologic pathways, particularly amongst monocytes (30, 31). Similarly, Guo et al., performing a scRNA-seq of two patients with severe COVID-19 pre- and post-treatment with tocilizumab, observed a reduced enrichment of inflammatory pathways as well as a reduced expression of IL-6 receptor related pathways genes in tocilizumab-treated cells. Moreover, the authors showed an enrichment in CD14 expression associated with the presence of non-inflammatory classical monocytes, in tocilizumab-treated cells (30, 31). All these findings, together with the available clinical data, support the belief that tocilizumab may be effective in reducing the monocytes-related inflammatory burden that results in the adverse outcomes of COVID-19.

In line with previously reports (6, 15, 32), in our cohort, on hospital admission, COVID-19 patients showed higher sCD163 plasmatic levels compared to HD, especially those who developed ARDS during hospitalization. These findings highlight the activation of the monocytic/macrophage system during COVID-19 pneumonia and underline how the evaluation of sCD163 plasmatic level could be a valuable predictive marker of severe disease in COVID-19 patients. These data are corroborated by the positive correlations between sCD163 plasmatic levels and absolute neutrophil count, and neutrophil-lymphocytes ratio as well as the negative correlation between sCD163 plasmatic levels and absolute lymphocytes count observed. Indeed, several authors showed that leukocytosis and an increase of neutrophil-lymphocytes ratio are associated with worsen outcome in COVID-19 pneumonia (33–36).

Considering all COVID-19 patients, the first main result of our study was a significant reduction in sCD163 plasmatic levels after seven days from hospitalization compared to the time of hospital admission without reaching HD plasmatic levels. To verify whether the reduction of sCD163 plasmatic levels observed depended on tocilizumab treatment, COVID-19 patients were stratified into two groups: TCZ and non-TCZ. On hospital admission, sCD163 plasmatic levels were comparable in both groups and each of them showed significantly higher sCD163 plasmatic levels compared to HD. However, during hospitalization the longitudinal evaluation of sCD163 plasmatic levels showed a significant reduction only in TCZ group. Moreover, in TCZ group it was observed that, after the treatment, sCD163 plasmatic levels were comparable with those of HD, supporting the hypothesis of a specific modulation of sCD163 plasmatic levels mediated by tocilizumab. These data suggest a role of tocilizumab in modulating sCD163 plasmatic levels and are in line with those of Hashimoto et al., in which a group of COVID-19 patients exhibited a reduction in serum levels of different inflammatory cytokines after tocilizumab administration (32).

The second main result was obtained stratifying TCZ group according to therapy response into R and NR groups. On hospital admission, no significant difference in sCD163 plasmatic levels was observed comparing the two groups. However, the longitudinal evaluation of sCD163 plasmatic levels showed a statistically significant reduction in both groups, independently of the outcome. These results show a tendency for tocilizumab to reduce sCD163 plasmatic levels. Thus, the negative outcome observed in NR group could be associated with factors that should be clarified, since no significative difference was found neither in demographic nor laboratory findings, although these were notably higher in NR group. Finally, in R group, the reduction observed seven days from hospitalization is steady after 30-45 days from discharge.

Our study suffers from the limitation to include a low sample size and the lack of evaluation of sCD163 plasmatic levels for all patients included in R group. Hence, further extensive studies are needed to validate our preliminary data and draw firm conclusions.

Overall, our study provides a detailed examination of sCD163 plasmatic levels evolution over time and, to the best of our knowledge it is one of the first that performs a careful longitudinal evaluation of the effect of tocilizumab on sCD163 plasmatic levels in COVID-19 patients.

It supports the hypothesis that sCD163 plays a significant role in eliciting an immune response in COVID-19 infected population and hence, it is also associated with the phenomenon of cytokine storm.

Therefore, tocilizumab therapy can be an effective method to control the heightened immune response and it has a substantial beneficial effect in majority of COVID-19 patients.

## Data Availability Statement

The raw data supporting the conclusions of this article will be made available by the authors, without undue reservation.

## Ethics Statement

The studies involving human participants were reviewed and approved by Ethic Committee Lazio 2 (protocol number 0080757/2020). The patients/participants provided their written informed consent to participate in this study.

## Author Contributions

RM, AC, and ML: designed the study. MZ, PN, ET, and MG: performed laboratory testing, analyzed data, performed statistical analysis, and wrote the manuscript. RM, AC, and PN: assisted in designing the study, performed laboratory testing, and analyzed data. MZ and ML: discussed results and critically revised the manuscript. RM, AC, PZ, VB, BK, and CB: provided clinical samples and clinical data. MZ, ML, FM, CM, VV, and MC discussed result, read, and revised the manuscript. All authors contributed to the article and approved the submitted version.

## Funding

This study received support from Sapienza, University of Rome: Ricerca Ateneo Sapienza Progetti Medi 2020 (protocol number 000326_20).

## Conflict of Interest

The authors declare that the research was conducted in the absence of any commercial or financial relationships that could be construed as a potential conflict of interest.

## Publisher’s Note

All claims expressed in this article are solely those of the authors and do not necessarily represent those of their affiliated organizations, or those of the publisher, the editors and the reviewers. Any product that may be evaluated in this article, or claim that may be made by its manufacturer, is not guaranteed or endorsed by the publisher.
